# Changes in the Nutrient Composition of Barley Grain (*Hordeum vulgare* L.) and of Morphological Fractions of Sprouts

**DOI:** 10.1155/2021/9968864

**Published:** 2021-07-20

**Authors:** Luis T. Ortiz, Susana Velasco, Jesús Treviño, Beatriz Jiménez, Almudena Rebolé

**Affiliations:** ^1^Departamento de Producción Animal Laboratorio de Agronomía, Facultad de Veterinaria, Universidad Complutense de Madrid, Avda. Puerta de Hierro S/n 28040, Madrid, Spain; ^2^Equinocol SL Colonia Herrero de Rosales 4, Cercedilla 28470, Madrid, Spain

## Abstract

The objectives of the current study were (1) to evaluate the effect of sprouting on protein, amino acids, fats, fatty acids, starch, total soluble carbohydrates, and *ß*-D-glucan content of barley grains and (2) to know the content of these nutrients in the morphological fractions of sprouts: green shoot, residual structure of sprouted grain (RSSG), residual structure of sprouted grain plus unsprouted grain (RSSG plus UG), and root fractions and to determine the proportion of each of these fractions (on fresh and dry basis) in the sprout biomass. Barley grain was sprouted in a commercial germination chamber for a period of 6 days. Raw grain was used as a control. Results showed that crude protein, ether extract, total soluble carbohydrates, and cellulose content increased, whereas starch and *ß*-D-glucan content decreased in sprouted when compared with the control grain. Amino acid and fatty acid profiles were also affected. Thus, aspartic acid, threonine, alanine, valine, isoleucine, lysine, and tryptophan content increased and only that of glutamic acid decreased after sprouting. Regarding fatty acids, an increase in the relative concentration of C18 : 0 and C18:3n-3 and a decrease in that of C18:1n-9 were detected. Partitioning of sprouted barley into three morphological component fractions showed that the residual structures of sprouted grains plus unsprouted grain fraction made up 82.9% and 93.6% of sprout biomass, on fresh and DM basis, respectively, and the remainder was provided by the root fraction, 10.3% and 3.2%, respectively, and by the green shoot fraction, 6.8% and 3.1%, respectively. The three morphological fractions differed in the content of the most analyzed nutrients.

## 1. Introduction

Sprouting or germination is a simple, inexpensive, and effective method for improving the nutritional value of seeds, particularly cereals and legume grains used in human diets. This technological method is also used to obtain green fodder for animal feeding. On the other hand, it must be taken into account that the sprouting process carried out in germinator chambers has not any negative environmental impact.

During sprouting, chemical changes occur in the composition of the seed due to the complex metabolic and physiological processes that start when the original seed comes out of its latency stage. Once sprouting is initiated, the cell wall polysaccharides are degraded, which allows enzymes to access the cell content. A variable proportion of carbohydrates, proteins, and lipids are degraded into more simple and available compounds such as sugars, free amino acids, and fatty acids, respectively [1–5]. Besides, the sprouting process may affect the concentration of minerals, vitamins, and phytochemical compounds such as polyphenols, phytic acid, enzyme inhibitors, and glucosinolates [6–11]. These changes in nutrient content during sprouting of seeds depend on the effect of factors including species and variety, humidity, temperature, light, availability of oxygen for aerobic respiration, and time of sprouting [1,12–15]. In general, sprouting seeds in germinator chambers is a controlled process under well-defined and reproducible environmental conditions, and therefore, the effect of the mentioned factors is well controlled.

Most reports found in the literature about the effect of sprouting on the nutrient composition of barley grains refer to grains sprouted in the dark during about 48 hours for malting purposes [16–18]. However, few and incomplete information is available on changes in the nutrient composition of barley sprouted in germinator chambers with continuous light. Under these conditions, a compact mat or carpet occupying all the surface of the sprouting tray is obtained due to the interweaving roots of sprouted grains. In this sprouting mat, three morphological layered fractions can be distinguished: the upper layer (green seedling fraction), intermediate layer (residual structure of sprouted grain plus unsprouted grain fraction, briefly as RSSG plus US fraction), and lower layer (root fraction). As far as we have noticed, data on the nutrient composition of these morphological fractions forming the sprouted barley mat are lacking.

The objectives of the current study were (1) to evaluate the effect of sprouting at an industrial commercial scale on protein, amino acids, fats, fatty acids, starch, total soluble carbohydrates, and *ß*-D-glucan content of barley grains and (2) to know the content of these nutrients in the morphological fractions of sprouts.

## 2. Materials and Methods

### 2.1. Plant Material and Sprouting

Barley grains (*Hordeum vulgare* L.) were acquired from a commercial supplier in Madrid (Spain), cleaned by hand, and soaked in tap water containing 0.07% sodium hypochlorite solution for 30 min to inhibit microbial growth, drained off, and soaked in tap water for 5 h. Imbibed grains were spread in plastic trays (0.60 × 0.40 × 0.07 m) and sprouted for 6 days in a germinator chamber for commercial use (Equinocol SL, Cercedilla, Madrid, Spain), with a size of 13.0 × 2.5 × 2.5 m, equipped with automatic sprayed irrigation and controlled temperature (20°C), relative humidity (80%), and continuous light. The experiment was conducted in tetraplicate. At the end of the sprouting period, four trays were chosen at random and representative samples (*ca*. 400 g) of sprouted barley were taken from each tray. Then, each of these four samples was divided into two subsamples. A group of four subsamples (replicates) was used for chemical analyses. The other group of four samples (replicates) was used for separating, by hand, the morphological fractions of sprouts (green shoot, RSSG plus UG, and root fractions), and the proportion of each of these fractions (on fresh and dry basis) in the sprout biomass was determined. Subsamples for analysis were freeze-dried (Liolabor 3 L-85-3264, Telstar SA, Terrasa, Spain), ground to pass through a 0.5 mm stainless screen with a cyclone mill (Fritsch Pulverisetter-14, Laborgeraetebau GmbH, Idar-Oberstein, Germany), and stored in air-tight containers at −24°C until analysis. These samples were analyzed in duplicate for moisture, crude protein, amino acids, crude fat, fatty acids, starch, total soluble carbohydrates, cellulose, and *ß*-D-Glucan. Four samples of raw barley grains were also ground and stored at the same condition than those for sprout samples to serve as a control. Since sprouting of barley grain was made in a germinator chamber under well-defined and reproducible controlled conditions, biological replicates were not deemed necessary.

### 2.2. Analytical Methods

All analyses were carried out in duplicate. Moisture, protein as crude protein (N *x* 6.25), and fat as ether extract were determined following the standard methods described by the Association of Official Analytical Chemists [19]. Ether extract was determined in 2 g dried sample with diethyl ether by Soxhlet extraction during 16 h, evaporating solvent to dryness in a rotatory evaporator. Amino acid analysis was performed by *o*-phtaldialdehyde precolumn derivatization [20] following the hydrolysis of samples with 6 N HCl at 110°C for 22 h in sealed evacuated tubes. Amino acids were measured using a Hewlett-Packard 1100 HPLC system (Agilent Technologies GmbH, Walbronn, Germany) equipped with a fluorescence detector and C-18 reversed phase column (Hypersil AA-ODS). Cystine was determined as cysteic acid [21] and tryptophan after alkaline hydrolysis [19]. For fatty acid analysis, aliquots of the ether extract were methylated with a mixture of boron trifluoride (in 10% methanol w/v), hexane, and methanol (35 : 20 : 45, v/v/v) [22]. Separation of fatty acid methyl esters was achieved on a glass capillary column (Tecknokroma SupraWax-280, length 60 m, id 0.25 mm, film thickness 0.15 *μ*m), attached to a Varian CP-3800 gas chromatograph (Varian Analytical Instruments, Walnut Creek, CA, USA), equipped with a split injector and a flame ionization detector.

Starch was quantified after its hydrolysis by *α*-amylase and amyloglocosidase enzymes using the Megazyme kit assay procedure K-TSTA 10/15 (Megazyme International, Ireland), with glucose released colorimetrically measured (Hitachi U-2000 spectrophotometer, Japan). Total soluble carbohydrates (TSCs) were determined by extraction with water and measured colorimetrically by the anthrone method [23]. Cellulose was determined according to the procedure of Goering and Van Soest [24]. The content of *ß*-D-glucan was determined by enzymatic hydrolysis using the Megazyme kit assay procedure K-BGLU 04/06 (Megazyme International, Ireland), with glucose released colorimetrically measured.

### 2.3. Statistical Analysis

Data obtained from this study were analyzed by the general linear model (GLM) procedure using the SAS Computer Software [25]. The average results were subjected to one-way analysis of variance (ANOVA). Differences between means were compared using the least significant difference (LSD) test at the 0.01 and 0.05 probability levels.

## 3. Results and Discussion

### 3.1. Sprout and Its Morphological Fractions

Data on the moisture content of the raw and sprouted barley grains as well as of the morphological fractions of sprouts are presented in Table 1. Moisture content was 9-fold higher in the sprouted than in the raw grain, a result that is in line with other published data [16,17]. This high moisture detected for sprouts in comparison to that of raw grain is attributable to the large uptake of water during the germination of seeds. A number of factors including size, seed coat permeability, chemical composition, and available water may affect water uptake by seeds during sprouting [1]. Data from Table 1 also show that the highest moisture content was observed in the root fraction (93.4%), followed by that in the green shoot fraction (89.8%) and in the RSSG plus UG fraction (75.8%). Regarding the relative contribution of each morphological fraction to sprout biomass, the results show that 82.9% was provided by the sprouted and unsprouted fraction, 10.3% by the root material, and 6.8% by the green shoot material. Expressing these data on DM weight basis, the contributions of the mentioned fractions were 93.6%, 3.2%, and 3.3%, respectively.

### 3.2. Protein and Amino Acids

Table 2 shows the results of protein (expressed as crude protein) and amino acid content in sprouted and raw barley grains. Protein content of sprouted barley was 38.6% higher (*P* < 0.01) compared to that of raw grain. This result is in accordance with the observations reported by other researchers for cereal grains [26], soybean seed [27], pea seed [11], and chickpea seed [9]. In contrast, decreases in protein during sprouting were reported for winged bean seeds [28] and for rice grain [29]. However, no significant change in the protein level of barley during sprouting was reported by Chung et al. [30]. These apparent contradictory results might be attributable to the effect of different factors including species and variety, seed availability, and environmental conditions during sprouting [1,14]. Data from Table 2 also show that there were marked (*P* < 0.01) differences in the protein content among the morphological fractions of sprouted barley. Comparatively, the protein content was 130% and 123% higher in the green shoot and root fractions, respectively, than in the RSSG plus UG fraction, whereas no significant (*P* > 0.05) differences in protein content were detected between the green shoot and root fractions.

Amino acid content in the raw and in the sprouted barley grain as well as in the morphological fractions appears in Table 2. There were significant differences between the raw and sprouted barley with respect to amino acid composition. Thus, of the 17 amino acids analyzed, the concentration (g kg^−1^ DM) of aspartic acid, threonine, alanine, valine, isoleucine, lysine, and tryptophan increased (*P* < 0.01 or *P* > 0.05) and only that of glutamic acid decreased (*P* > 0.05) during sprouting. These results agree with those reported by Chung et al. [30], who observed that, of the 14 individual amino acids monitored, the content of seven of them increased during sprouting and that of the remaining ones did not exhibit significant changes. The current results also showed that tryptophan and aspartic acid were the amino acids exhibiting the highest increase (75.0% and 72.7%, respectively) during sprouting, followed in decreasing order by lysine (48.5%) and alanine (41.3%). Dalby and Tsai [31] reported significant increases in the concentration of lysine and tryptophan during sprouting for 5 days of wheat, barley, triticale, rye, and oats.

With respect to the morphological fractions of sprouts, results showed that the content of most amino acids analyzed was higher in both the green and root fractions than in the RSSG plus UG fraction. The largest differences were found for histidine, glycine, and tryptophan content in the green shoot fraction and for histidine and glycine content in the root fraction which were 286, 156, and 154%, respectively, and 136 and 124% greater, respectively, compared to the content of these amino acids in the RSSG plus UG fraction. It was also noticeable that the high content of tryptophan (9.9 g kg^−1^ DM) was observed in the green shoot fraction compared to the root (4.9 g kg^−1^ DM) and RSSG plus UG (3.9 g kg^−1^ DM) fraction.

### 3.3. Fat and Fatty Acids

Fat content, expressed as ether extract, was 50.2% higher (*P* < 0.01) in the sprouted than in the raw barley grain (Table 3). Increases in the fat content during sprouting of barley were also reported by other researchers [16,17]. This rise in fat might be due to both an increase in the production of structural lipids associated with seedling growth and compositional changes occurring after degradation of other chemical constituents [16]. Concentration of fatty acids in the raw barley grain was also affected by the sprouting process (Table 3). The content of C18 : 3 n-3 and C18 : 0 increased by 49% and 24% (*P* < 0.01), respectively, and that of C18 : 1 n-9 decreased by 6% (*P* < 0.05) during the 6 days of sprouting. The concentration of linoleic acid, the predominant fatty acid in both the row and sprouted barley, was not affected (*P* > 0.05) by sprouting. These results are in accordance with those reported by Peer and Leeson [17]. Results from Table 3 also evidenced significant differences in the fat and fatty acid contents among the three morphological fractions of sprouted barley. Fat content was 43% and 31% higher (*P* < 0.01) in the green shoot and root fractions, respectively, than in the RSSG plus UG fraction, whereas no differences (*P* > 0.05) were detected between the values for the two firstly cited morphological fractions. Fatty acid composition of the three morphological fractions of sprouts was predominantly composed by polyunsaturated fatty acids (from 56.1 to 61.4%), followed in decreasing order by monounsaturated fatty acids and saturated fatty acids in the green shoot fraction and by saturated fatty acids and monounsaturated fatty acids in the RSSG plus UG fraction. In the case of the root fraction, the concentration of both the monounsaturated and saturated fatty acids was rather similar (*P* > 0.05). Regarding the concentration of fatty acids, the corresponding results showed that C18:2n-6 was the predominant fatty acid in the fat of the three morphological fractions. About the other major fatty acids, the concentration was the following in decreasing order: C18:3n-3, C18:1n-9, and C16 : 0 (24.8, 24.6, and 13.3%, respectively) in the green shoot fraction; C16 : 0, C18 : 1 n-9, and C18:3n-3 (19.5, 14.3, and 6.5%, respectively) in the RSSG plus UG fraction; and C18:1n-9, C16 : 0, and C18:3n-3 (19.6, 19.1, and 18.0%, respectively) in the root fraction. Palmitic acid was the most abundant of the saturated fatty acids in the three morphological fractions of sprouts.

### 3.4. Carbohydrates

Table 4 collects the content of starch, total soluble carbohydrates, and *ß*-D-glucan in both raw and sprouted barley grain. Sprouted barley exhibited a markedly (*P* < 0.01) lower starch content than raw grain, the amount decreasing by 18.5%. Reduced values of starch during sprouting cereal grains have been also reported by other researchers [1,32,33]. The decline in the starch content of starchy grains and seeds during sprouting is attributable to the fact that energy during sprouting and initial growth is mainly provided by the breakdown of starch as a result of an increased activity of starch-degrading enzymes. Thus, during steeping and sprouting of grains, three enzymes (*α*- and *ß*-amylase and *ß*-amyloglucosidase) hydrolyze the starch molecules into smaller compounds, mainly sugars, which are used as an energy source for the development of the embryo [34–36]. Besides, according to the work in [30], it is possible that the sprouting process may produce changes in the structure of starch molecules. On the other hand, the current results show that there were significant (*P* < 0.01) differences for starch values in the three morphological fractions of sprouts, and starch content was 506 and 111% higher, respectively, in the sprouted and RSSG plus UG fraction than in the green shoot and root fractions. In turn, starch content was 187% higher in the green shoot fraction than in the root fraction.

Total soluble carbohydrate content in contrast to the decreasing effect observed for starch was affected positively (*P* < 0.01) by the sprouting process of barley, and an increase of 245% was detected for these compounds. Other researchers [16] also detected higher levels of TSC content after sprouting barley grain. More recently, Shark et al. [37] determined the changes in the level of soluble carbohydrates during the germination and seedling establishment of barley and observed that such levels increased during the first days of sprouting and suffered a rapid decrease during the period from 9 to 12 days. Presumably, this is due, as mentioned above, to a partial degradation of starch into simple sugars during sprouting of seeds. As also shown in Table 4, TSC amount differed (*P* < 0.01) among the three morphological fractions of sprouted barley. Thus, TSC content was 24 and 28% higher in the green shoot fraction than in the RSSG plus UG fraction and in the root fraction, respectively. Differences between these latter fractions were also significant (*P* < 0.05), the content of TSC being 10% higher in the root fraction than in the RSSG plus UG fraction.

Cellulose content was also significantly (*P* < 0.01) influenced by sprouting of barley. The value of cellulose for raw barley grain was 27.4 g kg^−1^ DM, and after sprouting, that value increased by 188%. This result is in agreement with the findings of Fazaeli et al. [16], who reported that the acid detergent fibre content, an analytical fraction composed predominantly of cellulose, increased from 72.0 g kg^−1^ DM in raw barley grains to 143.5 g kg^−1^ DM in sprouted grains for 6 days. Martín-Cabrejas et al. [38] reported that sprouted peas (*Pisum sativum*) for 6 days exhibited significantly higher content of insoluble dietary fibre and higher proportion of cellulose compared to raw seeds. Likely, Masood et al. [9] reported that significantly higher values for crude fibre were observed in chick pea (*Cicer arietinum)* and mung bean (*Vigna radiate*) seeds for the entire period of sprouting (24, 48, 72, 96, and 120 hours) than in the corresponding unsprouted seeds. According to Peer and Leeson [17] and Cuddeford [39], the increase of fibre content during sprouting of seeds can be explained by the consumption of starch and by the increment in the synthesis of structural carbohydrates. On the other hand, the data concerning cellulose in Table 4 show that there were significant differences (*P* < 0.01) among the values obtained for the three morphological fractions of sprouted barley. The amount of cellulose was 21% higher in the green shoot fraction than in both the RSSG plus UG fraction and the root fraction. For these later morphological fractions, the values for cellulose content were rather similar.

The content of *ß*-D-glucan detected in barley grain was 32.0 g kg^−1^ DM, that is, at the middle of the range from 1.86 to 5.37% reported by Havrlentová and Kraic [40] as a result of analyzing 111 genotypes of barley. Of the different factors that may influence the level of *ß*-D-glucan in barley grains, genetic background seems to be the most important factor [32,41]. Results of the present research show that sprouting barley for 6 days caused a reduction of 50% (*P* < 0.01) in the *ß*-D-glucan content. Other researchers [32] reported a decline of 20.5% in *ß*-D-glucan content during sprouting barley for 48 h. This difference found for the effect of sprouting on the *ß*-D-glucan of barley might be explained because the sprouting period was 6 d in the current study and only 48 h in the study reported by the cited researchers. In any case, the decline in the *ß*-D-glucan content in barley grain during sprouting seems to be due to a mobilization of cell-wall-soluble polysaccharides and further breakdown into low-molecular compounds to be used as an energy source [42]. Regarding the composition of morphological fractions of sprouted barley, the current results show that the highest level of *ß*-D-glucan was found in the green shoot fraction (*P* < 0.01), and the level of *β*-D-glucan was 203 and 24% higher in the green shoot fraction than in the RSSG plus UG and in the root fractions, respectively. In turn, the *β*-D-glucan content in the root fraction was 40% higher than in the RSSG plus UG fraction.

## 4. Conclusions

Based on the results of the present study, it can be concluded that sprouting of barley grain led to increases in protein, fat, total soluble carbohydrate, and cellulose content and decreases in starch and *β*-D-glucan content. Pronounced changes in the amino acid and fatty acid profiles were also detected. Partitioning of sprouted barley into morphological fractions evidenced marked differences in the nutrient content among green shoot, RSGG plus UG, and root fractions. A high level of tryptophan was observed in the amino acid composition of the green shoot fraction of sprouts.

## Figures and Tables

**Table 1:** Moisture content in raw and sprouted barley grain and in morphological fractions of sprouts and proportion of morphological fractions of sprouts.

	Barley grain				
	Raw grain	Sprouted		Pooled SEM	Significance
Moisture (g kg^−1^)	88.5 ± 0.00	788.8 ± 1.06		3.75	^*∗∗*^

	Morphological fractions of sprouts				

	Green shoots	RSSG plus UG	Roots	Pooled SEM	Significance
Moisture (g kg^−1^)	897.5 ± 4.0^a^	758.3 ± 5.91^b^	934.2 ± 6.70^c^	2,83	^*∗∗*^

*Proportion in sprout biomass (*g kg^−1^)					

On fresh-matter basis	67.8 ± 9.21^a^	829.0 ± 11.29^b^	103.2 ± 6.28^c^	4.59	^*∗∗*^
On DM basis	32.5 ± 5.84^a^	935.8 ± 6.03^b^	31.7 ± 1.21^a^	2.45	^*∗∗*^

^a–c^Means with different superscripts within the same subheading and row are significantly different (^*∗∗*^*P* < 0.01). Values are means of 4 replicates (each replicate was a composite sample from 4 subsamples). RSSG: residual structures of sprouted grain: UG: unsprouted grain.

**Table 2:** Crude protein and amino acid content (g kg^−1^ DM basis) in raw and sprouted barley grain and in morphological fractions of sprouts.

	Barley grain					Morphological fractions of sprouted barley			
	Raw	Sprouted	Pooled SEM	Significance	Green shoots	RSSG plus UG	Roots	Pooled SEM	Significance
Crude protein	106.2 ± 1.47	147.2 ± 0.8	0.580	^*∗∗*^	313.8 ± 7.71^a^	136.6 ± 2.25^b^	304.9 ± 11.73^c^	4.120	^*∗∗*^

*Amino acids*									

Asp	7.7 ± 0.45	13.3 ± 1.28	0.48	^*∗∗*^	25.0 ± 1.47^a^	11.8 ± 1.50^b^	15.1 ± 1.02^c^	0.84	^*∗∗*^
Glu	24.7 ± 0.89	20.7 ± 2.10	0.81	^*∗*^	21.7 ± 1.54^a^	23.2 ± 1.95^ab^	26.4 ± 1.26^bc^	1.07	^*∗∗*^
Ser	5.0 ± 0.54	5.6 ± 0.76	0.33	NS	8.0 ± 0.66^a^	6.0 ± 0.56^b^	6.7 ± 0.55^b^	0.35	^*∗∗*^
His	2.4 ± 0.30	2.2 ± 0.56	0.19	NS	5.4 ± 1,03^a^	1.4 ± 0.34^b^	3.3 ± 1,27^c^	0.48	^*∗∗*^
Gly	4.4 ± 0.80	3.6 ± 0.97	0.45	NS	6.4 ± 1.30^a^	2.5 ± 0.0.6^b^	5.6 ± 0.42^a^	0.45	^*∗∗*^
Thr	4.0 ± 0.25	4.9 ± 0.37	0.16	^*∗∗*^	8.0 ± 0.78^a^	5.1 ± 0.51^b^	6.5 ± 0.62^c^	0.32	^*∗∗*^
Ala	4.6 ± 0.51	6.5 ± 0.79	0.33	^*∗∗*^	10.1 ± 0.88^a^	6.5 ± 0.59^b^	7.6 ± 0.39^b^	0.37	^*∗∗*^
Arg	5.8 ± 0.25	6.3 ± 0.90	0.33	NS	9.3 ± 1.07^a^	7.0 ± 0.72^b^	7.2 ± 0.27^b^	0.45	^*∗∗*^
Val	5.3 ± 0.08	6.4 ± 0.78	0.28	^*∗*^	9.9 ± 0.77^a^	6.0 ± 0.70^b^	9.7 ± 0.40^a^	0.34	^*∗∗*^
Met + Cys	4.9 ± 0.66	5.2 ± 0.46	0.28	NS	7.9 ± 1.36^a^	4.5 ± 0.60^b^	6.0 ± 0.68^b^	0.52	^*∗∗*^
Phe + Tyr	9.8 ± 0.57	11.0 ± 1.21	0.47	NS	14.8 ± 2.02^a^	6.6 ± 1.35^b^	11.1 ± 0.75^b^	0.77	^*∗∗*^
Ile	4.7 ± 0.92	6.3 ± 0.67	0.40	^*∗*^	8.7 ± 0.99^a^	5.4 ± 0.62^b^	8.1 ± 0.50^a^	0.37	^*∗∗*^
Leu	7.6 ± 0.47	8.2 ± 0.52	0.36	NS	12.0 ± 1.23^a^	9.0 ± 0.83^b^	12.0 ± 0.64^a^	0.52	^*∗∗*^
Lys	3.3 ± 0.40	4.9 ± 0.38	0.20	^*∗∗*^	8.6 ± 0.85^a^	5.9 ± 0.57^b^	7.5 ± 1.01^a^	0.47	^*∗∗*^
Trp	2.0 ± 0.22	3.5 ± 0.45	0.18	^*∗∗*^	9.9 ± 0.99^a^	3.9 ± 1.38^b^	4.9 ± 1.60^b^	0.67	^*∗∗*^

^a–c^Means with different superscripts within the same heading and row are significantly different. Data are means of 4 replicates (each replicate was a composite sample from 4 subsamples). ^*∗*^*P* < 0.05; ^*∗∗*^*P* < 0.01; NS: not significant. RSSG: residual structures of sprouted grain: UG: unsprouted grain.

**Table 3:** Crude fat content (g kg^−1^ DM basis) and fatty acid profile (g kg^−1^ total fatty acids) in raw and sprouted barley grain and in morphological fractions of sprouts.

	Barley grain				Morphological fractions of sprouted barley				
	Raw	Sprouted	Pooled SEM	Significance	Green shoots	RSSG plus UG	Roots	Pooled SEM	Significance
Crude fat	24.5 ± 0.00	36.8 ± 0.20	0.06	^*∗∗*^	48.4 ± 2.16^a^	33.9 ± 0.40^b^	44.5^a^ ± 2.78^a^	1.54	^*∗∗*^

*Fatty acids*									

C16:0	201.4 ± 1.50	199.7 ± 3.88	0.17	NS	133.3 ± 1.01^a^	195.4 ± 3.38^b^	191.5 ± 5.69^b^	2.19	^*∗∗*^
C18:0	16.5 ± 3.79	20.5 ± 0.86	0.04	^*∗∗*^	22.3 ± 1.01^a^	20.3 ± 1.37^a^	15.9 ± 1.96^b^	0.84	^*∗∗*^
C18:1n-9	151.0 ± 3.80	141.6 ± 4.15	0.22	^*∗∗*^	245.9 ± 6.35^a^	143.3 ± 3.22^a^	196.4 ± 23.78^b^	7.90	^*∗∗*^
C18:2n-6	566.5 ± 3.16	565.0 ± 8.04	0.35	NS	313.3 ± 7.67^a^	556.4 ± 1.26^a^	391.0 ± 2.35^b^	2.25	^*∗∗*^
C18:3n-3	45.2 ± 0.15	67.3 ± 4.28	0.18	^*∗∗*^	247.8 ± 4.91^a^	65.4 ± 2.22^c^	179.8 ± 9.76^b^	3.43	^*∗∗*^
C20:0	6.5 ± 3.03	4.5 ± 0.41	0.11	NS	2.8 ± 0.76	4.3 ± 0.51	3.1 ± 1.30	0.49	NS

^a–c^Means with different superscripts within the same heading and row are significantly different. Data are means of 4 replicates (each replicate was a composite sample from 4 subsamples). ^*∗*^*P* < 0.05; ^*∗∗*^*P* < 0.01; NS: not significant. RSSG: residual structures of sprouted grain: UG: unsprouted grain.

**Table 4:** Nonstructural and structural carbohydrate content (g kg^−1^ DM basis) in raw and sprouted barley grain and in morphological fractions of sprouts.

	Barley grain					Morphological fractions of sprouted barley			
	Raw	Sprouted	Pooled SEM	Significance	Green shoots	RSSG plus UG	Roots	Pooled SEM	Significance
Starch	576.0 ± 2.98	469.4 ± 21.14	7.55	^*∗∗*^	219.1 ± 8.22^a^	462.8 ± 2.99^a^	76.4 ± 7.20^b^	3.27	^*∗∗*^
TSC	70.2 ± 2.22	244.9 ± 4.16	1.66	^*∗∗*^	350.6 ± 6.57^a^	229.2 ± 6.48^b^	251.5 ± 6.10^c^	3.26	^*∗∗*^
Cellulose	27.4 ± 3.06	78.9 ± 3.88	3.62	^*∗∗*^	90.5 ± 6.57^a^	74.7 ± 3.88^b^	74.7 ± 3.08^b^	4.74	^*∗∗*^
*β*-D-Glucan	32.0 ± 1.16	16.3 ± 0.65	0.47	^*∗∗*^	44.3 ± 3.71^a^	14.6 ± 0.84^b^	35.7 ± 2.59^c^	1.32	^*∗∗*^

^a–c^Means with different superscripts within the same heading and row are significantly different. Data are means of 4 replicates (each replicate was a composite sample from 4 subsamples). ^*∗*^*P* < 0.05; ^*∗∗*^*P* < 0.01; NS: not significant. RSSG: residual structures of sprouted grain; UG: unsprouted grain; TSC: total soluble carbohydrate.

## Data Availability

The data used to support the findings of this study are included within the article.

## Abbreviations

DM: Dry matter
RSSG: Residual structure of sprouted grain
TSC: Total soluble carbohydrate
UG: Unsprouted grain.
